# Ectopic Expression in *Arabidopsis thaliana* of an NB-ARC Encoding Putative Disease Resistance Gene from Wild Chinese *Vitis pseudoreticulata* Enhances Resistance to Phytopathogenic Fungi and Bacteria

**DOI:** 10.3389/fpls.2015.01087

**Published:** 2015-12-10

**Authors:** Zhifeng Wen, Liping Yao, Ran Wan, Zhi Li, Chonghuai Liu, Xiping Wang

**Affiliations:** ^1^State Key Laboratory of Crop Stress Biology in Arid Areas, College of Horticulture, Northwest A&F UniversityYangling, China; ^2^Key Laboratory of Horticultural Plant Biology and Germplasm Innovation in Northwest China, Ministry of Agriculture, Northwest A&F UniversityYangling, China; ^3^Key Laboratory of Stress Physiology and Molecular Biology for Tree Fruits of Beijing, Department of Pomology, College of Agriculture and Biotechnology, China Agricultural UniversityBeijing, China; ^4^Zhengzhou Fruit Research Institute, Chinese Academy of Agricultural SciencesZhengzhou, China

**Keywords:** wild Chinese *Vitis*, *VpCN*, disease resistance, powdery mildew, promoter analysis

## Abstract

Plant resistance proteins mediate pathogen recognition and activate innate immune responses to restrict pathogen proliferation. One common feature of these proteins is an NB-ARC domain. In this study, we characterized a gene encoding a protein with an NB-ARC domain from wild Chinese grapevine *Vitis pseudoreticulata* accession “Baihe-35-1,” which was identified in a transcriptome analysis of the leaves following inoculation with *Erysiphe necator* (Schw.), a causal agent of powdery mildew. Transcript levels of this gene, designated *VpCN* (GenBank accession number KT265084), increased strongly after challenge of grapevine leaves with *E. necator*. The deduced amino acid sequence was predicted to contain an NB-ARC domain in the C-terminus and an RxCC-like domain similar to CC domain of Rx protein in the N-terminus. Ectopic expression of *VpCN* in *Arabidopsis thaliana* resulted in either a wild-type phenotype or a dwarf phenotype. The phenotypically normal transgenic *A. thaliana* showed enhance resistance to *A. thaliana* powdery mildew *Golovinomyces cichoracearum*, as well as to a virulent bacterial pathogen *Pseudomonas syringae* pv. tomato DC3000. Moreover, promoter::GUS (β-glucuronidase) analysis revealed that powdery mildew infection induced the promoter activity of *VpCN* in grapevine leaves. Finally, a promoter deletion analysis showed that TC rich repeat elements likely play an important role in the response to *E. necator* infection. Taken together, our results suggest that *VpCN* contribute to powdery mildew disease resistant in grapevine.

## Introduction

Plants have evolved multiple mechanisms to protect themselves against pathogens (Jones and Dangl, 2006). The first line of defense is microbe-associated molecular pattern (MAMP)-triggered immunity (MTI) following MAMP perception by membrane-resident pattern recognition receptors (Maekawa et al., 2011). MTI is thought to limit the growth of invasive pathogens. The second line of defense is plant innate immunity, which is activated by the specific recognition of pathogen-derived effectors by intracellular host resistance (R) proteins, and is termed effector-triggered immunity (ETI) (Chisholm et al., 2006). ETI typically leads to a hypersensitive response (HR) and gives rise to a faster and stronger defensive response than MTI-triggered immunity (Cesari et al., 2013). Understanding the function of R proteins, and the mechanisms by which they recognize pathogen effectors, can potentially lead to the development of a long-term strategy for the control and prevention of pathogen invasion.

Over the past few decades, numerous R genes have been cloned from model plants and important crops (Pan et al., 2000b; Collier and Moffett, 2009; Sekine et al., 2012). Most R proteins contain a nucleotide binding (NB) domain and a C-terminal leucine-rich repeat (LRR) domain, and belong to the so-called NB-LRR protein family (Ooijen et al., 2008). The most conserved domain in NB-LRR proteins is an NB domain that is found in proteins such as human Apaf-1, plant R proteins and *Caenorhabditis elegans* Ced-4 (ARC), and as such is referred to as the NB-ARC domain (Ooijen et al., 2008; van der Biezen and Jones, 1998). As a consequence of determining its three-dimensional structure, Albrecht and Takken (2006) proposed that the NB-ARC domain can be further divided into three sub-domains (NB, ARC1, and ARC2). Several conserved motifs have been identified thoughtout the NB-ARC domain in R proteins, such as Walker B, GxP, hhGRExE, Walker A or P-loop, MHD, and RNBS-A–D (Meyers et al., 1999; Pan et al., 2000a; Ooijen et al., 2008). Crystal structure analysis of the NB-ARC domain has led to the suggestion that it may function as a molecular switch to regulate signaling pathways through conformational changes (Riedl et al., 2005; Takken et al., 2006). It has also been shown that the nucleotide binding of the NB-ARC domain in the R proteins, I-2, and Mi-1, requires a P loop, since a P-loop mutant abolished the binding capacity (Tameling et al., 2010). Likewise, the oligomerization of an NB-ARC-LRR protein in the presence of its elicitor requires an intact P-loop in the NB-ARC domain (Mestre and Baulcombe, 2006).

Plant NB-LRR proteins can be divided into two distinct classes: the TNL and the CNL type, based on the domains present at their N terminus. Those that possess a Toll and human interleukin-1 receptor (TIR) domain are referred to as TIR-NB-ARC-LRR or TNL proteins, while those carrying a predicted coiled-coil (CC) domain are classified as CC-NB-ARC-LRR, or CNL proteins (Pan et al., 2000a; Lukasik-Shreepaathy et al., 2012). The potato (*Solanum tuberosum*) Rx protein is a typical CC-NB-ARC-LRR protein mediates resistance to potato virus X (PVX)(Kohm et al., 1993; Bendahmane et al., 1999), the CC domain of RX protein has a four bundle structure and forms a heterodimer with RanGAP2 WPP domain (Hao et al., 2013). The N-termini of the CC and TIR domains are thought to mediate downstream immune responses. It has been reported that in CNL proteins, the CC domain of NRG1 is capable of independently inducing defense responses (Collier et al., 2011), and in TIR proteins the TIR domain plays a crucial role in the cell death signaling pathway (Zhang et al., 2004; Weaver et al., 2006).

The identification and functional characterization of NB-ARC domain R proteins is of considerable interest in developing novel sources of disease resistance in crop plants that are threatened by phytopathogens. For example, *Erysiphe necator* is a fungus that causes powdery mildew (PM) disease in grapevine worldwide, resulting in serious losses in both grape yield and quality. The most economically important cultivated grapevine is *V. vinifera*, which is highly susceptible to PM (Gadoury et al., 2012). To combat the pathogen, fungicides are widely used, which causes environmental and financial pressure on grape growers and reduces wine quality. Thus, developing new grape cultivars with enhanced disease resistance mechanisms is of considerable interest. The wild Chinese *Vitis*, “Baihe-35-1,” is an accession of wild Chinese *V. pseudoreticulata* W. T. Wang that possesses high resistance to multiple fungi, and particularly to *E. necator* (Wang et al., 1995; Lin et al., 2006; Yu et al., 2011). To elucidate the resistance mechanisms involved in the defense response to fungal infection in this species, we previously performed an RNA-seq based transcriptome analysis *V. pseudoreticulata* “Baihe-35-1” that had been inoculated with *E. necator* (Weng et al., 2014). Among the pathogen induced genes, one was predicted to encode an NB-ARC domain protein.

In this current study, we report the isolation of the full length cDNA of this gene, which we designated *VpCN*, and its functional characterization following ectopic expression in *Arabidopsis thaliana*. Conclusions regarding its role in conferring Chinese Wild *V. pseudoreticulata* “Baihe-35-1” with disease resistance to powdery mildew are presented.

## Materials and methods

### Plant materials and growth conditions

Grapevines (Chinese wild *V. pseudoreticulata* accession Baihe-35-1 and *V. vinifera* cv. “Red globe”) were maintained in the grape germplasm resources orchard, Northwest A&F University, Yangling Shaanxi, China. *A. thaliana* (ecotype type, Columbia-0) was grown in a growth chamber under the following conditions: 22°C, 50% humidity, a 16/8 h day/night intensity of 125 μmolm^−2^ s^−1^ provided by cool white fluorescent bulbs.

### Cloning and sequence analysis

**Figure 1:**
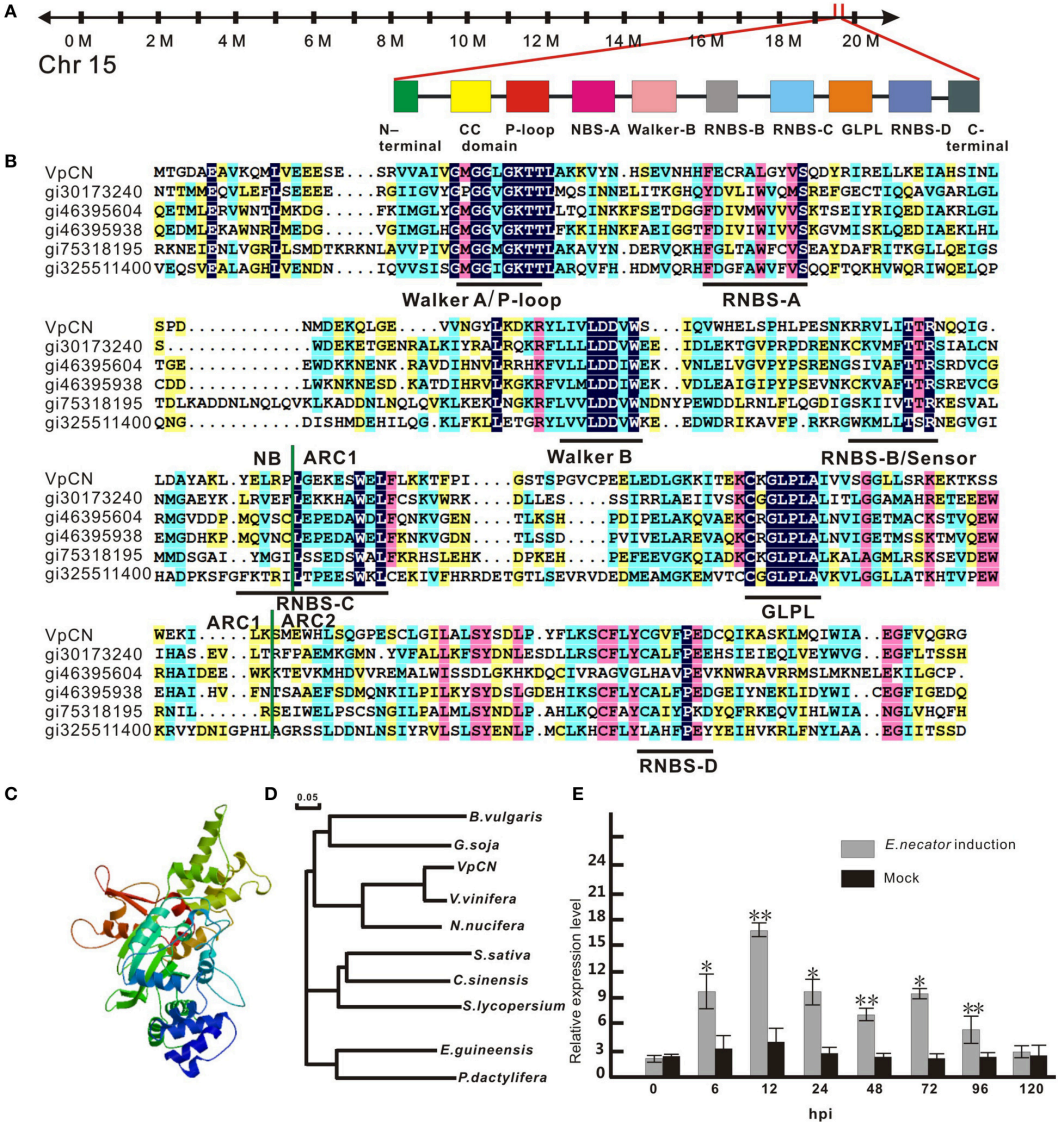
**Sequence analysis of *VpCN* and transcript level detection**. **(A)** Schematic map of *VpCN* location and major motifs. **(B)** Multiple sequence alignment of the NB, ARC1 and ARC2 subdomains of NB-ARC in VpCN with closely related proteins. Domain borders are indicate as vertical green lines. Motifs are labeled by horizontal dark lines below the aligned sequences. gi30173240 (Bent et al., 1994), gi46395604 (Bevan et al., 1998), gi46395938 (Theologis et al., 2000), gi75318159 (Ori et al., 1997), gi325511400 (Theologis et al., 2000) **(C)** Structural model of the NB-ARC domain of *VpCN*. **(D)** Phylogenetic tree of *VpCN* and related proteins from other plant species. The tree was generated using the ClustalW function in the MegAlign program: *Vitis vinifera* (GenBank accession no. XP010661747), *Nelumbo nucifera* (GenBank accession no. XP0102588251), *Glycine soja* (GenBank accession no. KHN19144), *Elaeis guineensis* (GenBank accession no. XP010913221), *Solanum lycopersicum* (GenBank accession no. XP010319316), *Beta vulgaris subsp. Vulgaris* (GenBank accession no. XP010669409), *Phoenix dactylifera* (GenBank accession no. XP008791188), *Camelina sativa* (GenBank accession no. XP010426119), *Citrus sinensis* (GenBank accession no. XP006470644). The scale bar represents 0.05 substitutions per site. **(D)** Structure model of NB-ARC in VpCN. **(E)** Analysis of *VpCN* expression in response to *E. necator* inoculation. The third to fifth fully expanded young grapevine leaves beneath the apex were selected for samples. The experiment encompass three independent biological replicates, for each biological replicate three leaves haversted from three plant and three technical replicates were performed. Data represent means of three biological replicates ±SE, asterisksin indicate statistical significance in comparison with control (Student's*t*-test, significance levels of ^*^P < 0.05, ^**^P < 0.01 are indicated).

Total RNA was extracted from grapevine as previously described (Zhang et al., 2003). First strand cDNA was synthesized from 1 μg of total RNA with the PrimerScript™ II 1st Strand cDNA Synthesis kit (TaKaRa Bio Inc., Dalian, China), according to the manufacturer's instructions. LA *Taq* (Takara Bio. Inc.) was used to amplify the ORF sequence of *VpCN*. The PCR products were cloned into the T-easy vector (Promega, USA), sequenced (Beijing Genomics Institute, Beijing, China) and submitted to GenBank (accession number KT265084). The *VpCN* cDNA sequence was analyzed using BLAST (http://Ncbi.nlm.Nih.gov/blast) in the NCBI database. Grapevine DNA extraction was conducted as previously described (Yu et al., 2013), primers for amplify promoter sequence were designed according to acquired sequence from Grape Genome Database (12 ×; http://www.genoscope.cns.fr), after cloning into the T-easy vector and sequencing, the promoter sequence was analyzed using PlantCARE (http://bioinformatics.psb.ugent.be/webtools/plantcare/html/) (Lescot et al., 2002). The deduced amino acid sequence of *VpCN* was aligned with closely related proteins and a phylogenetic tree was generated using neighbor joining algorithm with 1000 bootstrapping with the ClustalW tool in the MegAlign program (Version 5.07, DNASTAR Inc.) (Figure 1D). A structural model of the NB-ARC domain of *VpCN* was constructed using the structure of PDB 4m9x.1.C (Huang et al., 2013) in SWWISS-MODEL (Figure 1C). Real time PCR was conducted using SYBR^@^*Premix EX Taq*™II (Tli RNaseH Plus) (Takara Bio. Inc.) in a 20 μl volume reaction following the manufacturer's instructions using the CFX96TM real-time system (Bio-Rad, Hercules, CA, USA). The amplification cycles were as follows: initial denaturation at 94°C for 30 s, 40 cycles at 95°C 5 s, 60°C for 30 s. For melting curve analysis: 40 cycles at 95°C for 15 s followed by a constant increase from 60–95°C. The grapevine *Actin 1* (GenBank Accession number AY680701) was used as reference gene.

### Construction of vectors for ectopic expression and *A. thaliana* transformation

To generate *35S:VpCN*, the open reading frame (ORF) region of *VpCN* was cloned into the binary vector, pCAMBIA 2300 (CAMBIA company), downstream of the CaMV 35S promoter. The construct was introduced into *Agrobacterium tumefaciens*, strain GV3101, via electroporation, and the transformed *A. tumefaciens* was used to transform *A. thaliana* using the floral dip method (Clough and Bent, 1998). Transgenic plants were screened on MS (Murshige and Skoog, 1962) medium containing 60 mg/mL kanamycin, PCR amplification was performed to identify transgenic plants with gene specific primers.

### Construction of *VpCN* promoter:: *GUS* gene fusion vectors and *A. tumefaciens* mediated transient expression assays

To generate the *VpCN* promoter*:GUS* vector, the *VpCN* promoter was cloned into the T-easy vector, digested with *Bam*HI and *Pst*I, and finally cloned into the binary vector pC0380*GUS*. *35S:GUS* was used as a positive control (Xu et al., 2010). Four *pVpCN* promoter fragments with different 5′ deletions were amplified (Supplement Table 1). All the constructs were introduced into *A. tumefaciens* strain GV3101 via electroporation. The *A. tumefaciens* mediated transient expression assays were performed as previously described (Guan et al., 2011). *A. tumefaciens* GV3101 lines harboring the different constructs were grown in liquid Yeast Extract Phosphate (YEP) (Smith and Goodman, 1975) medium (supplemented with 100 μgml^−1^ kanamycin, 60 μgml^−1^ gentamycin, and 30 μgml^−1^ rifampicin) to an OD_600_ of 0.6, and harvested by centrifugation at 5000 × g for 10 min, before being resuspended in filtration solution (10 mM 2-(N-morpholino) ethanesulfonic acid (MES), pH 5.7, 10 mM MgCl_2_ and 15 μM acetosyringone) and adjusted to an OD_600_ of 0.6 for infiltration of young grapevine leaves using a vacuum infiltration method (Santos-Rosa et al., 2008). After infiltration, the leaves were kept in a chamber at 16/8 h day/night cycle at 23°C with 70% humidity for 48 h, before inoculation with *E. necator* (Guan et al., 2011; Yu et al., 2013).

### Pathogen inoculation procedures

*E. necator* infected leaves were collected from a highly PM-susceptible wild Chinese wild *V. Adstricta*, Hance clone “Taishan-2.” Leaves of the Chinese wild *V. pseudoreticulata* “Baihe-35-1” were inoculated by touching the adaxial epidermis of leaves with sporulating colonies on the surface of pathogen leaves, the inoculation were repeated three times (Guan et al., 2011). The samples were harvested 0, 6, 12, 24, 48, 72, 96, and 120 h after inoculation.

*A. thaliana* powdery mildew *G. cichoracearum* was maintained on highly susceptible *pad4 A. thaliana* mutant plants. The infection was conducted as previously described (Tang and Innes, 2002). The susceptibility or resistance phenotypes were scored 8 days after infection (Nie et al., 2011). Analyses of pathogenesis-related 1 (PR1) gene expression were performed using qRT-PCR using the same PCR program as for the *VpCN* analysis. The *A. thaliana* tubulin gene (GenBank Accession number NM_179953) was used as a reference. Rosett leaves from 4 week old *Arabidopsis* were harvested at 0, 12, 24, 36, and 48 h after inoculation.

*P. st* DC3000 cells grown in King's B medium (supplemented with 100 μgml^−1^ kanamycin and 30 μgml^−1^ rifampicin) to an OD_600_ of 0.6, harvested by centrifugation for 5000 × g for 10 min and re-suspended in 10 mM MgSO_4,_adjusted to optical density at OD_600_ of 0.02. The bacterial suspension containing 0.025% Silwet-77, and the mixture were hand infiltrated into the abaxial side of the *A. thaliana* leaves using a needless 1 ml syringe (Fan et al., 2008). *P. st DC*3000 bacterial growth were assessed 3 and 5 days after infection as described (Ahn et al., 2007).

### Trypan blue staining

For trypan blue staining, *A. thaliana* leaves were collected 12 hpi (hours post-inoculation) and boiled in alcoholic lactophenol trypan blue solution (20 mL of ethanol, 10 mL of phenol, 10 mL of water, 10 mL of lactic acid [83%], and 30 mg of trypan blue). Stained leaves were cleared in chloral hydrate (2.5 g dissolved in 1 mL of water) for 3 h, before placing under a coverslip in 50% glycerol (Koch and Slusarenko, 1990; Frye and Innes, 1998).

### Peroxide assay

Peroxide (H_2_O_2_) was assayed using a hydrogen peroxide kit, according to the manufacturer's instructions (Nanjing Bio Ins., Nanjing, China). Quantification of dead cells was performed 12 hpi by staining leaf discs (0.5 mm in diameter) with 0.2% Evans blue (Sigma) for 30 min, followed by several washes with water to remove excess stain (Mino et al., 2002; Ahn et al., 2007). One milliliter of 50% methanol supplemented with 1% SDS was added and the samples were incubated at 50°C for 1 h. Absorbance at OD_600_ was determined by ultraviolet spectrophotometry after a 10-fold dilution of the extracts (Ahn et al., 2007). The nitro blue terazolium (NBT) staining was performed as described (Kim et al., 2011).

### Callose accumulation

To observe callose accumulation, leaves (3 dpi) were immersed in destaining solution (10 ml phenol, 10 ml glycerin, 10 ml lactic acid, 10 ml H_2_O, and 80 ml ethanol) and kept in an oven at 60°C for 1 h to remove chlorophyll. The samples were washed to remove the destaining solution, and stained with 0.1% aniline. The fluorescence of callose was detected using an epifluorescence microscope (E800, Nikon) with a V-2A filter (Reuber et al., 1998; Ahn et al., 2007). For quantitative determination of callose, *A. thaliana*, leaves (3 dpi) were immersed in ethanol for 2–3 days to remove the chlorophyll, before centrifugation at 5000 × g for 10 min. The supernatant was discarded and the pellet resuspended in 0.4 ml DMSO. One hundred microliter of the supernatant was supplemented with loading mixture [400 μl 0.1% (w/v) aniline blue, 590 mL 1 M glycine/NaOH (pH 9.5), 210 mL 1 M HCl] and 200 μl 1 M NaOH. The control samples were not supplemented with aniline. The samples were incubated in a water bath 50°C for 20 min and cooled to room temperature before detection with a fluorescence spectrophotometer (F-4600, Hitachi, Tokyo, Japan) under 393 nm excitation, 479 nm emission and a voltage of 400 v. The fluorescence of the samples was determined by subtracting the fluorescence value of the control from those of the samples (Kohler et al., 2000).

### GUS staining, histochemical and fluorometric assays for determining *GUS* activity

A histochemical β-glucuronidase (GUS) assay of leaves was carried out as previously described (Jefferson, 1987). Briefly, leaves were immersed in GUS staining solution at 37°C for 24 h, before washing in 70% ethanol at 37°C and viewing macroscopically (Guan et al., 2011; Yu et al., 2013). GUS fluorescence was determined quantitatively according to Jefferson (1987). Protein concentrations in grapevine extracts was normalized by dilution with extraction buffer according to Bradford (1976). GUS activity was expressed as pmol 4MU (Sigma-Aldrich China, Shanghai, China) per minute per mg of protein. Sample fluorescence was detected with an infinite 200® PRO (Tecan Trading AG, Switzerland). Three independent experiments were performed.

## Results

### *VpCN* expression during powdery mildew infection

To identify potential resistance mechanisms and resistance related genes in the response of wild Chinese *V. pseudoreticulata* to powdery mildew, we previously performed a transcriptome analysis of the “Baihe-35-1” using RNA-seq (Weng et al., 2014). We observed that the expression of *VpCN* (GenBank accession number KT265084) was strongly induced by inoculation with *E. necator*. To verify this, we performed quantitative real-time PCR (qPCR) analysis of *VpCN* expression in *V. pseudoreticulata* leaves that had been inoculated with *E*. *necator*, and observed 4.2-fold greater *VpCN* transcript levels than in leaves prior to inoculation. Subsequently, *VpCN* expression decreased but remained at a higher level than in mock inoculated plants (Figure 1E).

### Cloning and sequence analysis of *VpCN*

To investigate the putative role of *VpCN* in providing resistance to pathogens, we first designed primers based on a cDNA sequence obtained from the Grape Genome Database (12 ×; http://www.genoscope.cns.fr), and isolated and designated the gene *VpCN* (GenBank accession number KT265084). The *VpCN* gene is located on chromosome 15 (Figure 1A), has an ORF of 1773 bp (Supplement Figure 1) and is predicted to encode a protein of 590 amino acids with a molecular mass of 67,390 Da and a theoretical pI value of 5.45. The amino acid sequence was further predicted to contain a RxCC-like domain in the N-terminus from residue 6–119, a Ran GTPase-acting protein 2 (RanGAP2) interaction site in the RxCC-like domain and an NB-ARC domain spanning residues 129–414. The NB-ARC sub-domains, NB, ARC1, and ARC2 were all present. Furthermore, several conserved motifs, such as a P-loop, RNBS A–D, and a GLPL (Figure 1B) were detected. In addition to a RxCC-like domain and an NB-ARC domain, we also found an AAA domain and a PLN03210 domain in the predicted amino acid sequence (picture not shown). A structure-based multiple amino acids sequence alignment was performed to compare the NB-ARC domain of *VpCN* with those of other closely related plant R proteins, including RPS2 (gi30173240) (Bent et al., 1994) and I-2 (gi75318159) (Ori et al., 1997). The amino acids sequence identity between the *VpCN* and the *A. thaliana* RPS2 NB-ARC domain was shown to be 33%, while the *VpCN* and I-2 NB-ARC domains had a 29%, sequence identity, concentrated on the conserved motifs of the NB-ARC subdomains (Figure 1B).

### Ectopic expression *VpCN* in *A. thaliana* enhance resistance to powdery mildew

**Figure 2:**
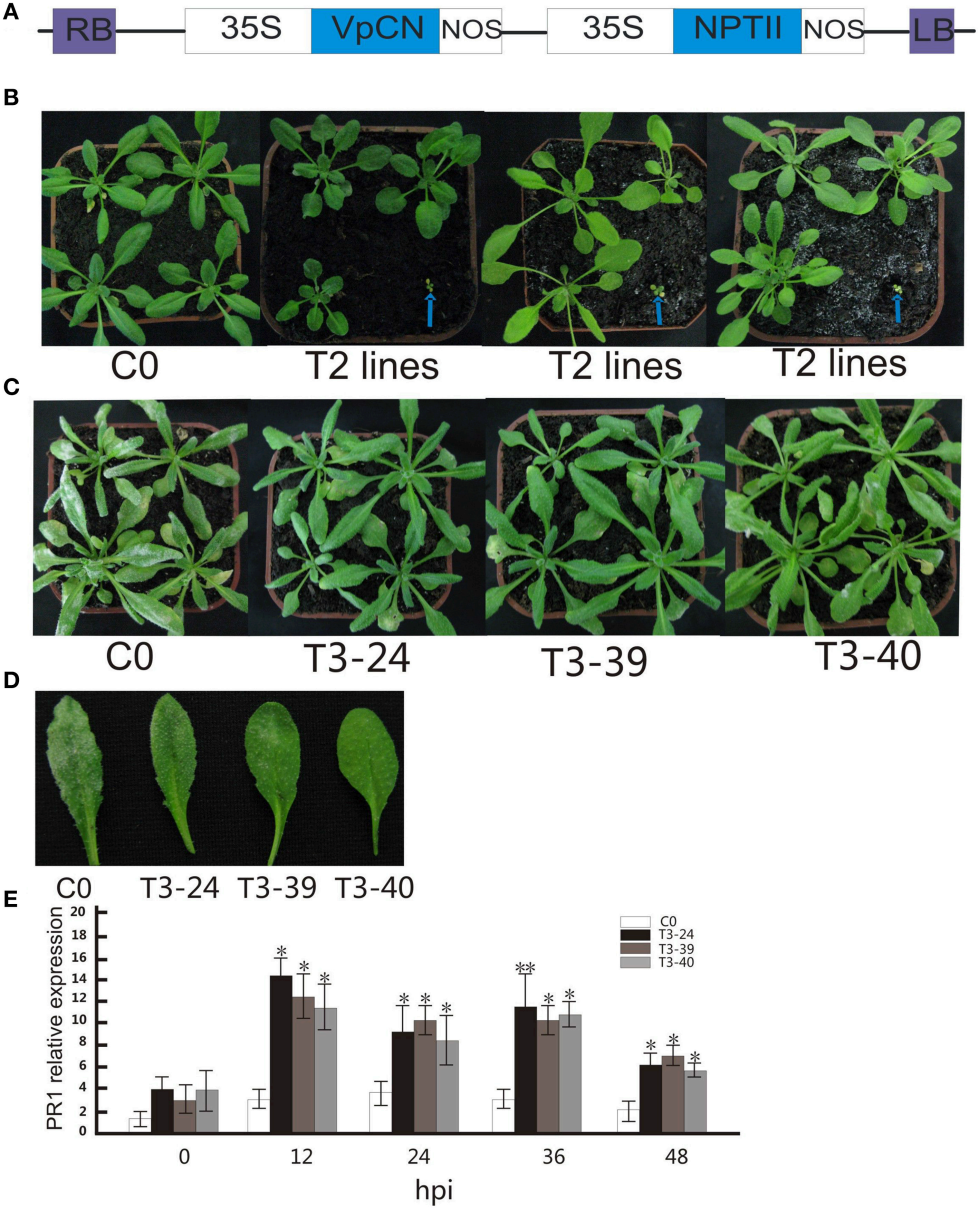
**Generation of CaMV 35S promoter-*VpCN* constructs used for transformation of *Arabidopsis thaliana*, morphology of wild type and transgenic *Arabidopsis thaliana* plants, with transgenic plants showing enhanced disease resistance to *G. cichoracearum* after ectopic expression of *VpCN***. **(A)** Structure of the CaMV 35S promoter-*VpCN* ectopic expression construct. LB, left border; RB, right border; 35S, CaMV 35S promoter; NOS, terminator; NPT II, aminoglycoside-3′- phosphotransferase. **(B)** Indicate T2 transgenic plants displayed either normal phenotypes or dwarfism. Blue arrows indicate the dwarf phenotype in 4 week old plants. **(C)** Transgenic *A. thaliana* leaves developed fungal spores 8 dpi with *G. cichoracearum*. **(D)** Disease symptoms developed on the leaves of transgenic lines and wild type plants 8 dpi with *G. cichoracearum*. **(E)** *A. thaliana* PR1 transcript levels in T3 lines and wild-type after inoculation with *G. cichoracearum*. Total RNA was extracted from *A. thaliana* leaves 0, 12, 24, 36, and 48 h post-inoculation (hpi) with *G. Cichoracearum*. The experiment encompass three independent biological replicates, for each biological replicate six rosette leaves were harvested from three plant and three technical replicates were performed. Data represent means of three biological replicates ±SE, asterisksin indicate statistical significance in comparison with WT (Student's *t*-test, significance levels of ^*^P < 0.05, ^**^P < 0.01 are indicated).

We next transformed the *VpCN* in *A. thaliana* under the control of the constitutive 35S promoter (Figure 2A). A total of 42 independent transgenic T1 lines were obtained and the presence of the transgene confirmed by PCR using *VpCN* specific primers. The T2 progeny segregated so that 39 lines displayed wild type morphology while three lines exhibited a dwarfed phenotype and morphological abnormalities, such as small yellow leaves, stunted growth, and chlorotic tissue (Figure 2B). These dwarf lines eventually died. The lines with a wild type phenotype were challenged with *G. cichoracearum*, and three transgenic lines with higher resistance were chosen for the generation of homozygous T3 generation lines. The transgenic lines displayed few visible white powdery areas on their leaves at 8 dpi, whereas the wild-type (Col-0) exhibited abundant powdery mildew development (Figures 2C,D). To determine whether the enhanced resistance to *G. cichoracearum* in the transgenic lines was related to an increase in the expression of a known defense gene, we evaluated PR1 (Pathogenesis Related 1) (Friedrich et al., 1996) transcript levels at 0, 12, 24, 36, and 48 hpi. Three transgenic plants displayed higher PR1 transcript abundance after pathogen inoculation than wild type plants, reaching a maximum level at 12 hpi. The PR1 transcript levels of transgenic plants were ~4–5-fold higher after inoculation than in wild type at all time points (Figure 2E).

### Ectopic expression of *VpCN* results in enhanced protection against the bacterial pathogen, *P. st* DC3000

**Figure 3:**
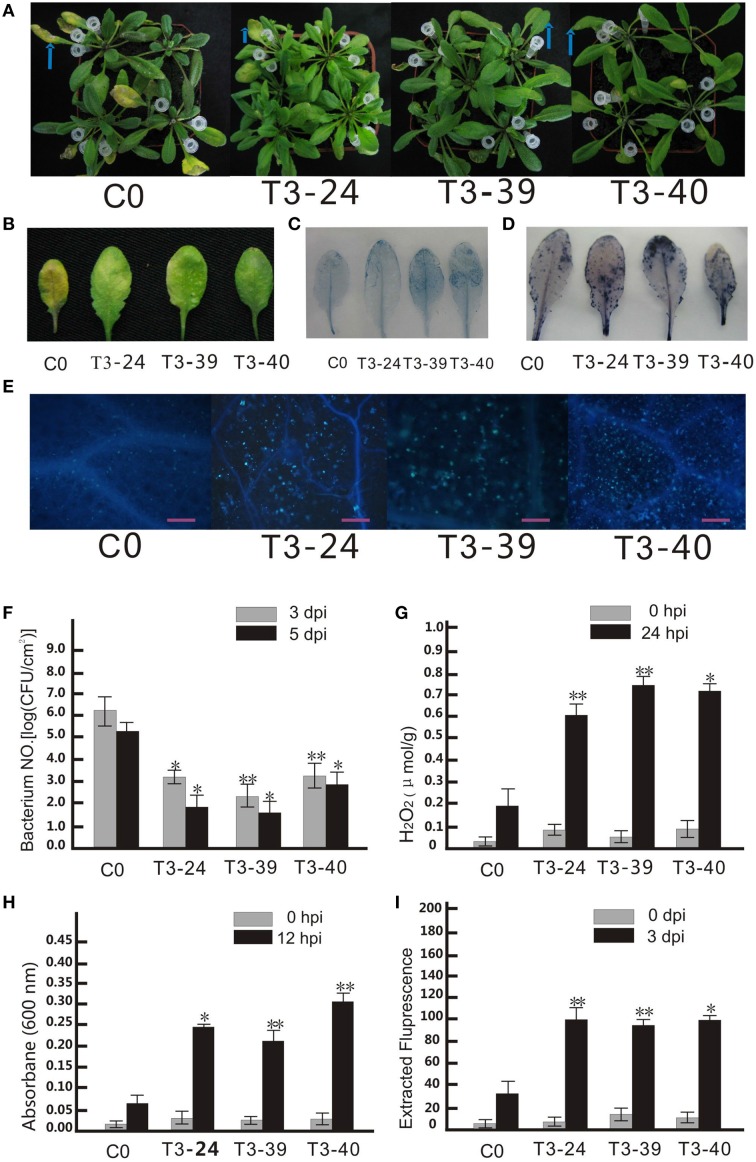
**Ectopic expression of *VpCN*in *Arabidopsis thaliana* enhanced disease resistance to *Pseudomonas syringae* pv. tomato DC3000**. **(A)** *P. st* DC3000 was diluted to OD_600_ 0.02 and injected into the middle of a leaf with needleless syringes. The injected leaves were marked with white pipette tips, and pictures taken 3 dpi. **(B)** Disease symptoms developed on the leaves of transgenic lines and wild type plants 3 dpi with *P.st* DC3000. **(C)** Transgenic plants and wild type leaves were stained with trypan blue 12 hpi with *P.st* DC3000. **(D)** Transgenic plants and wild type leaves were stained with nitro blue terazolium (NBT). **(E)** Microscopic observation of callose deposition after 3 dpi. Bars = 50 μm. **(F)** The numbers of bacterial cells in the leaves were determined at 3 and 5 dpi. **(G)** Detection of H_2_O_2_ concentration in *Arabidopsis* leaf samples harvested at 24 hpi. **(H)** Quantification of dead cells at 12 hpi. **(I)** Quantification of callose from *A. thaliana* leaves at 3 dpi. The experiment encompass three independent biological replicates, for each biological replicate six rosette leaves were harvested from three plant and three technical replicates were performed. Data represent means of three biological replicates ±SE, asterisksin indicate statistical significance in comparison with WT (Student's *t*-test, significance levels of ^*^P < 0.05, ^**^P < 0.01 are indicated).

Since amino acid sequence of *VpCN* was predicted to contain a PLN03210 domain, which has been shown to be correlated with resistance to *Pseudomonas syringae pv. glycinea* race 6 (Kim et al., 2009), we hypothesized that it might function in providing resistance to bacterial infection. To test this, transgenic and control plants were challenged with the bacterial *P. st* DC3000 pathogen by leaf infiltration (Figure 3A). Most infiltrated wild type leaves exhibited water-soaking at 1 dpi, turned yellow and finally wilted at 5 dpi. In contrast, the transgenic plants infected with the pathogen showed fewer symptoms (Figure 3B), and when the growth of *P. st* DC3000 in the inoculated plants was quantified, it was found that the bacterial number in the transgenic plants was significantly lower than in the wild type plants (Figure 3F). To observe the effect of *VpCN* expression on cell death, trypan blue staining was performed of leaves and we observed that cell death was more widespread in the transgenic lines than the wild type plants (Figure 3C). Additionally, cell death quantification by Evans blue staining followed by spectrophotometric analysis, showed a 5-6 fold higher level cell death in the transgenic plants (Figure 3H). Nitroblue tetrazolium (NBT) staining for the superoxide anion also showed higher accumulation in the transgenic plants (Figure 3D), as did quantitative measurements of H_2_O_2_ (Figure 3G). Finally, the accumulation of the (1,3)-β-glucan polymer callose, which is known to be involved in plant defense responses (Brown et al., 1998), was visualized by aniline blue staining of wild type and transgenic plants after treated with *P. st* DC3000 (Figure 3E). Greater accumulation of callose was observed in the transgenic plants than in wild, and when callose levels were quantified, it was confirmed that the transgenic lines contained significantly (*P* < 0.05) more callose (Figure 3I).

### Isolation and analysis of the *VpCN* promoter sequence

**Figure 4:**
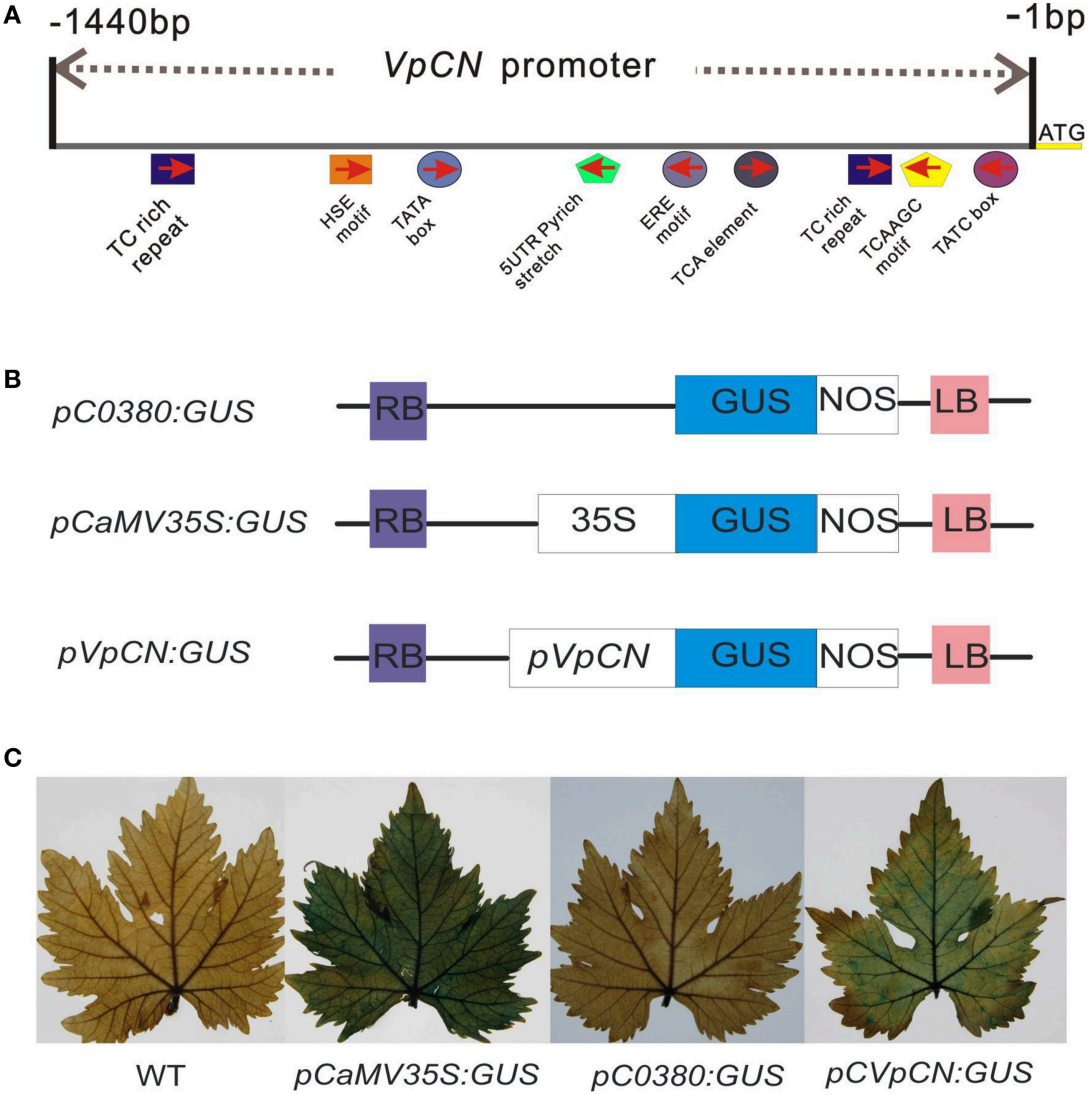
**The main predicted *cis*-acting elements in the *pVpCN* promoter sequence, structure of the *VpCN* promoter fused to the *GUS* reporter gene and GUS staining of the transient constructs in transformed grapevine leaves**. **(A)** Schematic diagram of the main predicted *cis*-acting elements in the *VpCN* promoter sequence of Chinese wild *V. pseudoreticulata*. **(B)** The *pVpCN* promoter was fused to the *GUS* gene. The plasmid pCaMV35S:GUS was used as a positive control and pC0380:GUS was used as a negative control. **(C)** The fully expanded grapevine leaves of *V. vinifera* “Red globe” were collect from a grape germplasm resources orchard and used for agroinfiltration.

A 1440 bp upstream sequence was cloned using wild Chinese *V. pseudoreticulata “*Baihe-35-1” genomic DNA by PCR, regulatory *cis*-acting elements predicted showed that several putative regulatory elements involved in the activation of defense-related genes, including 72 predicted TATA boxes, 32 CAAT boxes, and two TC-repeat elements, which are known to be involved in defense and stress responses, a TCA element, which is involved in salicylic acid (SA) responses, a TGACG motif, which is associate with methyl jasmonate-response, an HSE element, which is involved in heat stress responses and two TATC elements, which are related to gibberellin responses (Figure 4A). Additional predicted *cis*-regulatory elements included light response elements (TCCC-motif, MRE, I-box, GT1-motif, GAG-motif, GA-motif, G-box, CATT motif, Box-I, AT1-motif, and Box-4), as well as others *cis*-elements (5UTR Py-rich stretch, circadian element and, TATC box). Several of the predicted *cis*-elements are known to be involved in responses to environmental stresses, further suggesting that the *VpCN* promoter may play a role in defense responses.

### Promoter::GUS (glucuronidase) assays

**Figure 5:**
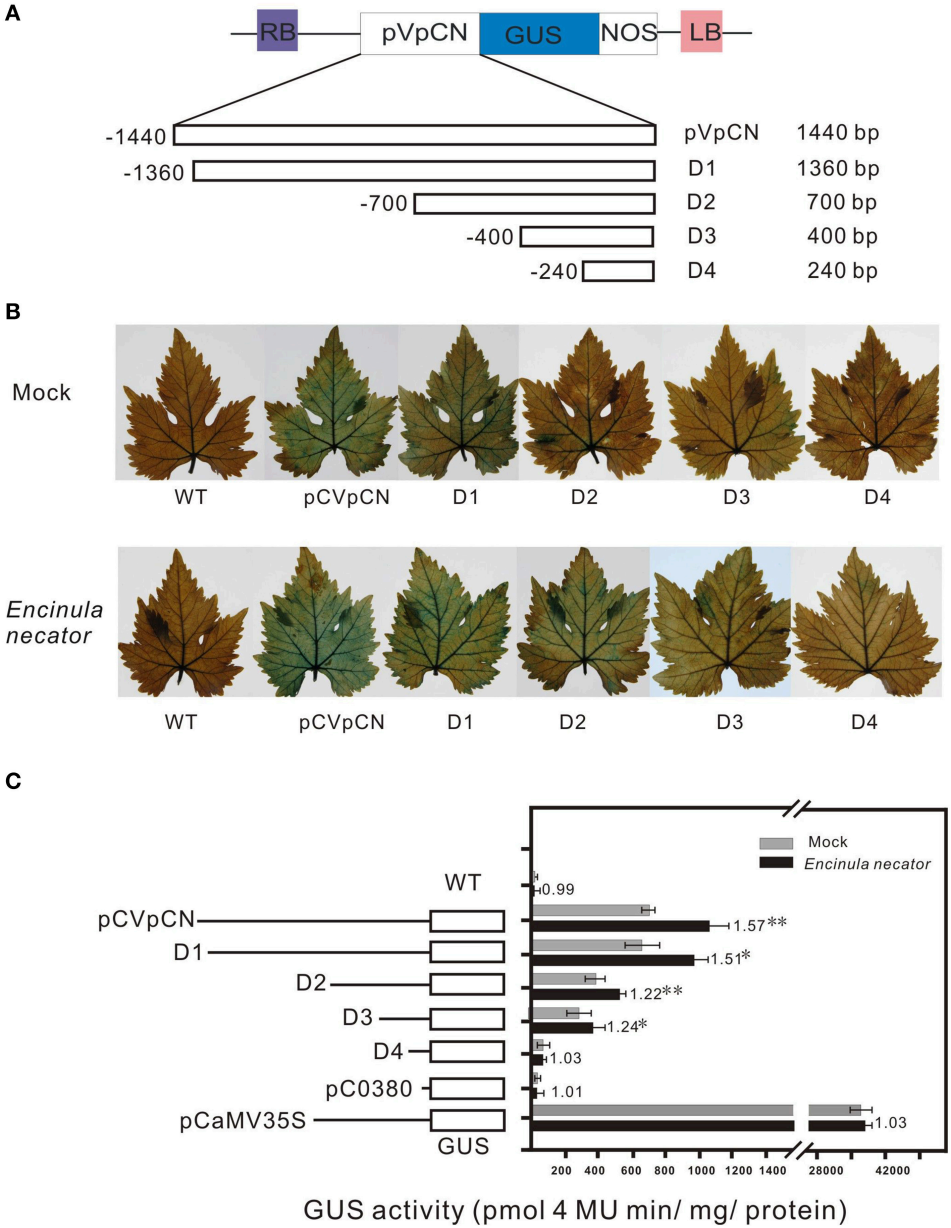
**Schematic map of the p*VpCN* promoter-*GUS* gene fusion deletion constructs, histochemical analysis of GUS expression in transiently transformed *V. vinifera* “Red globe” leaves after inoculation with *E.necator*, and fluorometric analysis of GUS activity in the transiently transformed grapevine leaves**. **(A)** The *GUS* gene was driven by the *VpCN* promoter deletions, the exact locations of the promoter fragments are shown in Supplement Figure 2. The deletion size is indicated at the far right. **(B)** GUS staining was carried out 2 days after treatment with sterile water (upper panel) or *E. necator* (lower panel). **(C)** The various deletion fragments of the *VpCN* promoter fused to *GUS* and relative GUS activity driven in the transiently transformed grapevine leaves. The *dark bars* indicate the average *GUS* activity for deletion constructs in transiently transformed grapevine leaves treated with *E. necator*, the *gray bars* indicate the mock treatment (sterile water). Numbers adjacent to the bars indicate the fold difference in GUS activity leaves harboring the various constructs challenged with *E. necator* relative to the mock samples. The mean GUS activity (±SD) is averaged from three independent experiments (*n* = 3), the errors bars indicate the stand deviation. Significant difference between treatment and mock conditions was analyzed using one sided paired *t*-test (^**^ and ^*^ meaning *P* < 0.0.1 or *P* < 0.05, respectively).

To test the activity of the *VpCN* promoter, the 1440-bp promoter fragment was fused to a reporter gene encoding β-glucuronidase (GUS), generating the construct pCVpCNGUS. As a positive control, a CaMV35S::GUS (PC35SGUS) construct was used and a construct with no promoter was used as a negative control (pC0380GUS) (Xu et al., 2010; Figure 4B). All the constructs were expressed transiently in grapevine leaves, which were subsequently subjected to GUS staining. Leaves transformed with the PC35SGUS construct showed strong GUS activity, while no activity was detected in wild type (WT) and very little in PC0380GUS. pCVpCNGUS transformed leaves showed GUS activity but at a lower level than leaves transformed with PC35SGUS (Figure 4C), and when leaves were infected with *E. necator* 2 dpi prior to GUS staining, the infected leaves exhibited stronger GUS activity than mock-inoculated control leaves. To further determine the location of the pathogen-responsive *cis*-regulatory region, we generated four promoter deletion fragments and fused them to *GUS* (−1360, −700, −400, and −240 bp) (Figure 5A). When the GUS activity was quantified fluorescently, the highest levels were measured in grapevines containing the −1440 bp fragment, where it was induced 1.57-fold after treatment with *E. necator* compared to mock controls. Leaves transformed with −1360, −700, and, −400 promoter fragments exhibited a relative low level of GUS activity; however, they showed increased GUS activity after being challenged with *E. necator* (Figure 5B). Since the leaves transformed with the −240 bp fragment showed no significant difference in GUS activity before and after treatment with *E. necator* (Figure 5C), the −400 bp promoter fragment was deduced to be the minimal promoter region required for the response to *E. necator* infection.

## Discussion

We previously reported the leaf transcriptome of wild Chinese grape (*V. Pseudoreticulata*, “Baihe-35-1”) that had been inoculated with *E. necator*, and showed that expression of a unigene corresponding to *VpCN* was strongly induced by the infection (Weng et al., 2014). Here, we isolated the ORF sequence of *VpCN* and ectopically expressed it in *A. thaliana*. This resulted in enhanced disease resistance to the pathogens *G. cichoracearum* and *P. st* DC3000. The deduced amino acid sequence of the corresponding protein is predicted to contain an RxCC-like and an NB-ARC domain. Most currently known R proteins have a NB-ARC domain and the CC domain is thought to initiate signaling (Radirdan et al., 2008). Given the rapid and strong up-regulation of *VpCN* transcript accumulation in wild Chinese *Vitis* after treatment with *E. necator*, we suggest that *VpCN* may play a role in the early defense signaling pathways in pathogen recognition. In addition to these two domains, the deduced amino acid sequence also contained a PLN03210 domain, which is thought to contribute to the identification of resistance signaling components and to convey resistance to *P. syringae* (Kim et al., 2009), suggesting that *VpCN* may also be associated with bacterial disease resistance.

Several studies have already demonstrated that over-expression of an R-gene can cause growth retardation, spontaneous cell death, and constitutive defense activation (Tao et al., 2000; Bendahmane et al., 2002; Stokes et al., 2002; Mohr et al., 2010; Nandety et al., 2013) due an over activation of the ETI system. In this study, three independent transgenic lines exhibited dwarfism and stunted growth, as well as other morphological defects, although since these plants eventually died, we were unable to investigate whether they also exhibited enhanced resistance to *G. Cichoracearum*. In agree with these results we suggest that *VpCN* ectopic expression may active ETI system and cause constitutive defense in three transgenic plants and cause growth retardation, spontaneous cell death. Further studies will investigate whether the three dwarf and lethal phenotypes is caused by toxic effects of high level of *VpCN* expression or the co-suppression between *VpCN* and *Arabidopsis* endogenous genes with VpCN-homologous sequences.

There have been several reports suggesting that over-expression of R genes enhances disease resistance due to constitutive SA accumulation, PR gene expression and active defense responses (Keller et al., 1999; Tang et al., 1999; Kim et al., 2001; Shirano et al., 2002; Stokes et al., 2002). In this study, ectopic expression of *VpCN* in *A. thaliana* enhanced disease resistance to *G. cichoracearum*, and when the PR1 transcript levels was assessed, a 4-5 fold increase in expression was observed in 12 hpi in transgenic plants compared to WT, and these levels remained higher over the time course. These results suggest that ectopic expression of *VpCN* in *A. thaliana* activate defense responses after pathogen inoculation.

The production of reactive oxygen species (ROS), mainly in the form of a superoxide burst and H_2_O_2_ accumulation, is thought to enhance plant defense responses and to be essential for the establishment of plant immunity (Alvarez et al., 1998; Grant and Loake, 2000; Punja, 2004; Choi and Hwang, 2011; Kim and Hwang, 2014). In agreement with these results, we found that higher levels of ${\text{O}}_{2}^{-}$ anions and H_2_O_2_ in the transgenic plants than in WT after challenging with *P. st* DC3000. This suggests that ectopic expression of *VpCN* triggers an oxidative burst to induce plant immunity to *P. st* DC3000; however, further studies are needed to investigate how oxidative burst and H_2_O_2_ accumulation is mediated by *VpCN*. High concentrations of ROS can result in HR-like cell death (Kovtun et al., 2000; Wang et al., 2007; Zhang et al., 2012), and over-expression of a TIR-NB-LRR gene from wild north American grapevine in *V. vinifera* wine grape cultivars was reported to lead to HR-like cell death after inoculation with *E. necator* (Feechan et al., 2013). Moreover, over-expression of a RPP1A truncation in *A. thaliana* induced elicitor-independent HR-like cell death (Weaver et al., 2006). In this study, an increase in ROS (${\text{O}}_{2}^{-}$ and H_2_O_2_) accumulation followed by H_2_O_2_ induced HR-like cell death was observed after ectopic expression of *VpCN* in *A. thaliana*, when the transgenic plants were inoculated with *P. st* DC3000.

Callose-containing cell-wall appositions, called papillae, provide a physical barrier that slows pathogen invasion at the site of pathogen attack (Luna et al., 2011). Callose deposition is typically triggered by conserved pathogen-associated molecular patterns (PAMPs) and contributes to the innate immunity (Brown et al., 1998; Luna et al., 2011). Ellinger et al. (2013) demonstrated that over-expression of *PMR4* in transgenic plants promoted early callose accumulation at attempted fungal penetration sites, which provided complete resistance to *G. cichoracearum*, and the non-adapted PM agent, *B. graminis*. In this study, transgenic plants displayed more callose deposition than WT plants in response to treatment with *P. st* DC3000, suggesting that callose deposition may contribute to the enhanced disease resistance to the pathogen displayed by the transgenic plants.

To elucidate the molecular basis of *VpCN* transcript induction after inoculation with *E. necator*, the *VpCN* promoter was isolated and its activation investigated using *A. tumefaciens*-mediated transient expression of *VpCN* in *V. vinifera* leaves. Bioinformatic analysis of the promoter sequence revealed two TC-rich repeats (′5-ATTCTCTAAC-3′), which are thought to be involved in defense and stress responses (Diaz-De-Leon et al., 1993). We hypothesized that these might be involved in the response to *E. necator*, and generated four promoter deletion constructs to test this idea. Plants harboring a −1360, −700, or −400 bp region of the promoter sequence, all of which contain two or one TC rich repeat elements (Supplement Figure 2), showed increased GUS activity after challenge with *E. necator*. However, plants containing only a −240 bp region sequence, which has no TC-rich repeat elements (Supplement Figure 2), showed no significant change in GUS activity after inoculation with *E. necator*. Thus, we propose that the TC-rich repeat elements may play a role in the *VpCN* promoter activity in response to *E. necator* infection. This study suggests that *VpCN* is a disease resistance gene, and we will investigate that whether the *VpCN* is interact with AVR protein (effector) from *Erysiphe necator*. Further functional studies to the VpCN with other proteins and downstream defense signaling involved in the powdery mildew disease resistance will be helpful in understanding the molecular mechanisms of powdery mildew disease resistance in Chinese wild *V. pseudoreticulata*.

## Author contributions

XW and ZW designed the experiments. ZW, LY, RW, and ZL performed the experiments. XW, ZW, and CL analyzed the results and wrote the manuscript. All authors read and approved the final manuscript.

### Conflict of interest statement

The authors declare that the research was conducted in the absence of any commercial or financial relationships that could be construed as a potential conflict of interest.

## Supplementary material

Supplement Table 1 **List of primer sequence used in this study**. F, Forward primer; R, Reverse primer.

Supplement Figure 1 **Sequence analysis *VpCN* from Chinese wild *V. pseudoreticulata* W. T. Wang “Baihe-35-1.”** The ORF sequence of *VpCN* is 1773 bp and encodes a polypeptide of 590 amino acids. The Rx-CC-like domain is labeled by single underline and the NB-ARC domain by a double underline.

Supplement Figure 2 **Sequence analysis of the *VpCN* promoter**. Motifs with significant similarity to previously identified *cis*-acting elements are shaded and the names are given under each element. Sequences labeled in yellow correspond to primer design positions. Arrow heads represent the start point of the 5-deleted promoter derivatives.
