# Supplementary material for: Ectopic Expression in Arabidopsis thaliana of an NB-ARC Encoding Putative Disease Resistance Gene from Wild Chinese Vitis pseudoreticulata Enhances Resistance to Phytopathogenic Fungi and Bacteria
Source: Front Plant Sci. 2015 Dec 10;6:1087. doi: 10.3389/fpls.2015.01087 (PMC4674559; doi:10.3389/fpls.2015.01087)
Supplement: Supplement Table 1 — List of primer sequence used in this study. F, Forward primer; R, Reverse primer. [file Table1.DOC]

| Primer name | oligonucleotide primers | Purpose or vector |
| --- | --- | --- |
| VpCN-PCR-F  VpCN-PCR-R | CGGGATCCCGATGGCGGACGGCAATATTACGTT  GGGGTACCCCTTAATTTATGGAGGTGATACCAC | pCAMBIA2300 |
| PCVpCN-PCR-F  PCVpCN-PCR-R | CGGATCCGATACATTAGGGCAAACTTATCATTC  GCTGCAGCTCTTTTAGTTACTGAAATTGTCTTC | PC0380GUS |
| VpCN-qRT- PCR-F  VpCN-qRT-PCR-R | GGGCAAGTGAGGCTGCTGCG  TCCGGCCATCAAGCTCATGGAT | qRT- PCR |
| VpActin-qRT- PCR-F  VpActin-qRT- PCR-R | GATTCTGGTGATGGTGTGAGT  GACAATTTCCCGTTCAGCAGT | qRT- PCR |
| PR1-qRT-PCR-F  PR1-qRT-PCR-R | GGAGCTACGCAGAACAACTAAGA  CCCACGAGGATCATAGTTGCAACTGA | qRT- PCR |
| AtTui-qRT-PCR  AtTui-qRT-PCR-R | TCAATCCAGGAGATGTTTAGG  ACTGCTGGTACTCTGCGACA | qRT- PCR |
| PCVpCN-PCR-F-D1 | CGGATCCGTCAATCCGTATACTCATATATAAT | PC0380GUS |
| PCVpCN-PCR-F D2 | CGGATCCGTGTCACACATTGGACAAACCTATC | PC0380GUS |
| PCVpCN-PCR-F D3 | CGGATCCGGGAATGAAGAAAGGAATGCCTTGA | PC0380GUS |
| PCVpCN-PCR-F D4 | CGGATCCGAGGCCGAGAGCCATTTCCACTTG | PC0380GUS |
