# Supplementary material for: Ectopic Expression in Arabidopsis thaliana of an NB-ARC Encoding Putative Disease Resistance Gene from Wild Chinese Vitis pseudoreticulata Enhances Resistance to Phytopathogenic Fungi and Bacteria
Source: Front Plant Sci. 2015 Dec 10;6:1087. doi: 10.3389/fpls.2015.01087 (PMC4674559; doi:10.3389/fpls.2015.01087)
Supplement: Supplement Figure 1 — Sequence analysis VpCN from Chinese wild V. pseudoreticulata W. T. Wang “Baihe-35-1.” The ORF sequence of VpCN is 1773 bp and encodes a polypeptide of 590 amino acids. The Rx-CC-like domain is labeled by single underline and the NB-ARC domain by a double underline. [file Table2.DOC]

1 ATGGCGGACGGCAATATTACGTTTTTTCTGGAGAAGTTAGGTAACTTTGTTGTACAAGAA

M A D G N I T F F L E K L G N F V V Q E

61 GCTTCCCTCTTCGGAGAGGTTGAAGGGCAAGTGAGGCTGCTGCGAAATGAGATGGAGTGG

A S L F G E V E G Q V R L L R N E M E W

121 ATGCGCCTGGTCCTGGAAGATGCAGATATTGATACAAAATGCAACCACGACAGAAGACTC

M R L V L E D A D I D T K C N H D R R L

181 AAGCTGTGGGTGAATCAGATCAGGGACGTAGCTTATGATGCTGAAGATGTCATCGATGAG

K L W V N Q I R D V A Y D A E D V I D E

241 TTCATGTTCAAGATTGAACACCAACGACAGCGGAGACCCAATCGGTTCTTGCCCACGTGC

F M F K I E H Q R Q R R P N R F L P T C

301 GTGAGATTTGCTGACAAGTTACCATTTATCCATGAGCTTGATGGCCGGATAAGAGAGATC

V R F A D K L P F I H E L D G R I R E I

361AATATCACAATTGAGAAGATTCTGGCTAATAAAGACAGGTACAACATCGAAAGTGGAATC

N I T I E K I L A N K D R Y N I E S G I

421 CCTTCTGAAGCTGGGAGCTCTTCCAGTACTGAAGGGGTGGTGCAAAGAGGGAAAAGGGTT

P S E A G S S S S T E G V V Q R G K R V

481 CCAATTGTTGAGGAAGCGGACGTAGTGGGCATGACAGGGGATGCGGAAGCAGTGAAGCAA

P I V E E A D V V G M T G D A E A V K Q

541 ATGCTGGTGGAGGAAGAGTCAGAGAGCAGAGTGGTGGCCATCGTTGGGATGGGTGGCCTT

M L V E E E S E S R V V A I V G M G G L

601 GGCAAAACTACTCTTGCTAAGAAAGTCTACAACCACAGTG AAGTTAACCA CCACTTTGAA

G K T T L A K K V Y N H S E V N H H F E

661 TGTCGCGCTTTGGGGTATGTATCTCAAGATTATAGAATCCGGGAGCTTCTGAAGGAGATA

C R A L G Y V S Q D Y R I R E L L K E I

721 GCCCACAGCATCAATCTCAGCCCTGATAATATGGATGAGAAACAGTTGGGGGAGGTGGTT

A H S I N L S P D N M D E K Q L G E V V

781 AATGGTTACCTCAAAGATAAAAGGTATTTGATAGTGTTGGATGATGTATGGAGCATCCAA

N G Y L K D K R Y L I V L D D V W S I Q

841 GTTTGGCATGAATTGAGTCCGCATCTTCCTGAGTCAAACAAGAGAAGAGTGCTCATCACT

V W H E L S P H L P E S N K R R V L I T

901 ACACGCAACCAACAAATTGGTTTGGATGCCTATGCAAAGCTCTATGAACTTCGTCCTTTA

T R N Q Q I G L D A Y A K L Y E L R P L

961 GGTGAAAAAGAGAGTTGGGAGCTCTTTCTCAAGAAAACTTTTCCTATTGGAAGTACATCA

G E K E S W E L F L K K T F P I G S T S

1021CCAGGTGTATGTCCTGAAGAGTTGGAAGATCTGGGAAAGAAGATTACAGAGAAATGCAAA

P G V C P E E L E D L G K K I T E K C K

1081GGCTTGCCTCTAGCCATTGTGGTATCAGGAGGGCTTCTATCAAGAAAAGAGAAGACGAAA

G L P L A I V V S G G L L S R K E K T K

1141 TCTTCATGGGAGAAAATACTTAAAAGCATGGAGTGGCATCTAAGTCAAGGCCCCGAGTCA

S S W E K I L K S M E W H L S Q G P E S

1201 TGCTTGGGAATTCTTGCTTTGAGTTATAGTGACTTGCCTTACTTCTTGAAGTCTTGCTTT

C L G I L A L S Y S D L P Y F L K S C F

1261 CTCTACTGCGGTGTTTTTCCGGAGGACTGCCAAATTAAGGCAAGCAAATTGATGCAGATA

L Y C G V F P E D C Q I K A S K L M Q I

1321 TGGATTGCAGAGGGATTTGTACAAGGAAGGGGTGAAGAAATGGTGGAAGACGTAGCTGAA

W I A E G F V Q G R G E E M V E D V A E

1381 GAGTATTTAGAAGAGCTGATTCACGGAAGCATGATTCAGGTGGCCGGAAGAAAATGGGAT

E Y L E E L I H G S M I Q V A G R K W D

1441 GGAAGAGTGAAGTCTTGTCGCATCCACGATCTGCTTCGTGACCTCGCTATTTCAAAAGCC

G R V K S C R I H D L L R D L A I S K A

1501AGAGATTCAAAAATTTTTGAGCTCGAATTTCTAAGGTTGAAGGAGTTGCCTGCATTAGAG

R D S K I F E L E F L R L K E L P A L E

1561 GAGTTGAAAGTGGAGGACGGAGCAATGCCTACTTTGAAAACTTTACAAATTGTTCACTGT

E L K V E D G A M P T L K T L Q I V H C

1621 AATGGGATGAAAACGTTGCTTCATGAGTTGTTGAAATTAAAAAATCCTCAACGAGTAAAT

N G M K T L L H E L L K L K N P Q R V N

1681 CTAGAAAGCATGAACGGTGAATTAATTCAAGAGATTGAAACCACAGAGGGAGAAGAATTC

L E S M N G E L I Q E I E T T E G E E F

1741 GACAAGATTCGTGGTATCACCTCCATAAATTAA

D K I R G I T S I N A *
