# Supplementary material for: Ectopic Expression in Arabidopsis thaliana of an NB-ARC Encoding Putative Disease Resistance Gene from Wild Chinese Vitis pseudoreticulata Enhances Resistance to Phytopathogenic Fungi and Bacteria
Source: Front Plant Sci. 2015 Dec 10;6:1087. doi: 10.3389/fpls.2015.01087 (PMC4674559; doi:10.3389/fpls.2015.01087)
Supplement: Supplement Figure 2 — Sequence analysis of the VpCN promoter. Motifs with significant similarity to previously identified cis-acting elements are shaded and the names are given under each element. Sequences labeled in yellow correspond to primer design positions. Arrow heads represent the start point of the 5-deleted promoter derivatives. [file Table3.DOC]

-1440 ATACATTAGGGCAAACTTATCATTCAATAAATAAAAAACAACCCCCTACATCTCCTACAG

-1380 AGAGAAATAGAATTAACTCAAACTTTATAATCAATCCGTATACTCATATATAATATAAAA

-1320 GTGTTTAAAATATTCTTCATATTTTTAATTATTTCTTAAAATATTTAATAAAAAAATATT

*TC rich repeats*

-1260 TAAAAATATCTAAAATTTATTTTAAAAATAACATGTGTTCAAAAGAAATTCTTAAAAGCA

-1200 ATTTTAAGTCAAAAATTTGTGAAGAGTATATTTTAAATCAAAATTTTTAATTAAAAAAAT

*HSE*

-1140 TTCAAAAATAGCCCCAAAGAATATTATTGATCATTAGACATTATTAGAATCCATTGTTTA

*ERE*

-1080 GACTTGTTTTTTTTTTTTTTTTTTTAAATTTTCTACTTCCTTATTTAATAAAAATGACCA

-1020 TTCCATTGAAGTACATGTGAAACTAAATAACAGCCCACAACACAATTAAGAGATGACAAA

-960 AGCCCTGGGGCCCATGTTGTTATGGAGTCATGGACCATATCGCAGCCCTGAACAATTGAT

-900 TTGCTTTGCTTTTACCTTAGTTTAGGTTACATGTGATTCCCAAAAACTACTATAGAAAGA

-840 AAAATAAATATTTAGAAAACAATTATTTTATATTTAAATACTCTAAATATTTACATTAAT

-780 TTCTTCCTTAATTCTACAAATCATGGATAATGAATTGGCTAATGAATTTGAGAGGTGTCA

-720 AGGCGGTTTTGTCACACATTGGACAAACCTATCTTGATCCAAATCTCTGTTCCACCACTT

-660 AGAAGAGCCAAATGCTTCCATGAAAATTATTCAAAGCTGAATTCAGTTAAAAACTAAAAA

*MBS*

-600 TTAAATTAAATTTAAAATAAGGGTTTCATGGGCCCGAATGAAGTAACAATGCTTCAAGAA

-540 AAGCACCTGGGATAACTTCAATCTGCCTCTCAAGTATTATACTTTTGTTTTACCTCTTGC

*TATA box*

-480 AAAACCCCATTATCCAAAAACAATGCAGAGAGCATCCATGTGATTGTTCTTAACCTTAAA

420 GAAAAGGATAATATAGGAATGAAGAAAGGAATGCCTTGAGAATGAAGAGAAGAAAGGGCA

*TCA element*

*5' UTR Py-rich strech*

-360 TGGATATATATAGAGATTTTGTGGGCAGAGAAGATCATACCCAGCCTTAATTTCTTCCTC

-300 GTCAAAGGCCGAGAGCCATTTCCACTTGTATTTTTGTTAATATTTTCTCCCTTCAGACCT

*Box III*

*TC rich repeats*

-240 CCAGTCAGATTGTTCCAGTCATTTCCATTATATCCTTATATGCTTTTTCCTTCATTTACC

-180 GGCCAATTGACTTCTGGGATATCTTCATCTGGCTCTTCTCCAGGAAGCCATTAGCATAGC

*TATC box*

-120 ATACCAACCTCTTGCCTCTCCACTACTTCCTAATTTTCCTCTTTTTCTTTGTCCGTTTTC

-60 GTTTGTTCAATATTCTCTCTTGTTTGTCATTGGAAGAAGACAATTTCAGTAACTAAAAGA

0 ATGGCGGACGGCAATATTACGTTTTTTCTGGAGAAGTTGGGCAACCTGGTTGTACAAGAA

GCTTCCCTCTTGGGAGAGGTTGAAGGGCAAGTGAGGCTGCTGCGAAATGAGATGGA

GTGGATGCGCCTGGTCCT
